# Accumulation of Inertial Sensory Information in the Perception of Whole Body Yaw Rotation

**DOI:** 10.1371/journal.pone.0170497

**Published:** 2017-01-26

**Authors:** Alessandro Nesti, Ksander de Winkel, Heinrich H. Bülthoff

**Affiliations:** Department of Human Perception, Cognition and Action, Max Planck Institute for Biological Cybernetics, Tübingen, Germany; VU University Amsterdam, NETHERLANDS

## Abstract

While moving through the environment, our central nervous system accumulates sensory information over time to provide an estimate of our self-motion, allowing for completing crucial tasks such as maintaining balance. However, little is known on how the duration of the motion stimuli influences our performances in a self-motion discrimination task. Here we study the human ability to discriminate intensities of sinusoidal (0.5 Hz) self-rotations around the vertical axis (yaw) for four different stimulus durations (1, 2, 3 and 5 s) in darkness. In a typical trial, participants experienced two consecutive rotations of equal duration and different peak amplitude, and reported the one perceived as stronger. For each stimulus duration, we determined the smallest detectable change in stimulus intensity (differential threshold) for a reference velocity of 15 deg/s. Results indicate that differential thresholds decrease with stimulus duration and asymptotically converge to a constant, positive value. This suggests that the central nervous system accumulates sensory information on self-motion over time, resulting in improved discrimination performances. Observed trends in differential thresholds are consistent with predictions based on a drift diffusion model with leaky integration of sensory evidence.

## Introduction

Everyday life requires humans to move through the environment, while completing crucial tasks such as maintaining balance or controlling a vehicle. Success in these tasks largely relies on a veridical perception of self-motion, i.e., the continuous estimation of one’s body position, and its derivatives, with respect to the world. This estimation process is performed by the central nervous system (CNS) by combining visual, auditory and inertial (i.e., somatosensory and vestibular) sensory information–seemingly without effort. Whereas a considerable body of neurophysiological and behavioural studies address how information on self-motion is accumulated across the senses (see e.g., [1–10]), much less is known about how information on self-motion is accumulated over time. Given the dynamic nature of natural self-movements, it is rather intuitive that the CNS must accumulate sensory information not only across the senses, but also over time. For instance, it has been shown that humans walking on a straight path in darkness can estimate their travelled distance, suggesting a path integration mechanism that continuously updates based on sensory information [11,12]. Nevertheless, the perceptual processes underlying the accumulation of sensory evidence, and in specific the effect of stimulus exposure time on the human ability to perceive and discriminate self-motion, remains largely unexplored.

In the present work, we employ a psychophysical approach to investigate whether the human ability to discriminate among different rotation intensities around the head vertical axis improves as a function of the time available for accumulating sensory information.

### Differential thresholds

Among the most common experimental paradigms in psychophysics is the two-interval two-alternative forced choice (2IFC) task [13]. In a 2IFC task, every experimental trial consists of two consecutive stimulus presentations, for which participants perform a relative comparison (e.g., report the stronger of the two motions). This allows to measure the smallest change in motion intensity that can be detected by a human observer in a given percentage of observations [13], i.e., the differential threshold (DT).

Experimentally measuring DTs has been a powerful tool for the study of different aspects related to self-motion perception, such as the relationship between physical and perceived motion intensity [6,14–18] or the processes underlying multisensory integration [1,4–6,9]. However, to the best of our knowledge, it remains an open question how DTs are affected by the stimulus duration, a relationship that might shed light on how sensory evidence in self-motion is accumulated by the CNS over time.

#### Drift diffusion model

Ratcliff [19] developed the Drift Diffusion Model (DDM) as a general framework to account for accumulation of evidence. This model has been employed, sometimes with slight variations, to predict reaction times and accuracy in a broad variety of psychophysical experiments (see e.g., [19–22]; for an overview on DDMs, see [23,24]). In general, the DDM relies on the basic assumption that information is accumulated continuously. The process of accumulating evidence is described by the position of a particle that drifts over time while also being subjected to noise. The drift rate determines the average speed at which information from the physical stimulus is accumulated. The noise reflects the probabilistic nature of perception, that is, the inter-trial variability that can lead to different responses to repetitions of the same stimulus. DDMs have also been successfully employed in recent neurophysiological works on decision making, which report neural correlates of accumulation of visual and auditory information in both monkeys [21,25] and humans [26,27].

To the best of our knowledge, the only study so far that directly investigated whether sensory information on self-motion is accumulated over time to the benefit of intensity discrimination was conducted by Drugowitsch and colleagues [22]. In this study, a psychophysical approach was used to measure reaction times with a one-interval two-alternative forced choice task (2AFC): participants were provided at every trial with visual-inertial cues of a linear motion and had to discriminate their heading direction (left or right). The study reported improved discrimination performances for longer reaction times and proposed a variation of the DDM that well described the trade-off between reaction times and accuracy.

### Present work

Here, we investigate whether the human ability to discriminate different motion intensities is affected by the time of exposure to the motion stimuli. Specifically, we hypothesize that DTs for the perception of head-centred yaw rotations in darkness improve (i.e., decrease) with increasing stimulus duration, due to the CNS ability to accumulate sensory evidence. Furthermore, we expect DTs to asymptotically converge to a constant, positive value, indicative of a source of perceptual noise independent from stimulus duration. We eventually propose two variations of the Ratcliff’s DDM to account for any effect of motion duration.

To avoid confounds due to the frequency-dependent nature of yaw perceptual thresholds, which in humans decrease for increasing frequencies until approximately 1 Hz, (see e.g., [28–30]), all stimuli employed in this study are sinusoidal with a frequency of 0.5 Hz and durations that are a multiple of 1 s. We employ supra-threshold stimuli to ensure that sensory evidence is available to the decision process throughout the entire stimulus.

## Methods

### Participants

Ten participants (age 24–36, 4 females), 8 naïve and 2 experimenters (AN and KW) took part in the study. None of them reported any history of balance or spinal disorders, nor motion sickness susceptibility. Participants gave their written informed consent (as outlined in PLOS consent form) prior to inclusion in the study, in accordance with the ethical standards specified by the 1964 Declaration of Helsinki. The experiment was approved by the ethical commission of the medical faculty of the Eberhard Karls University in Tübingen, Germany.

### Setup

**Fig 1:**
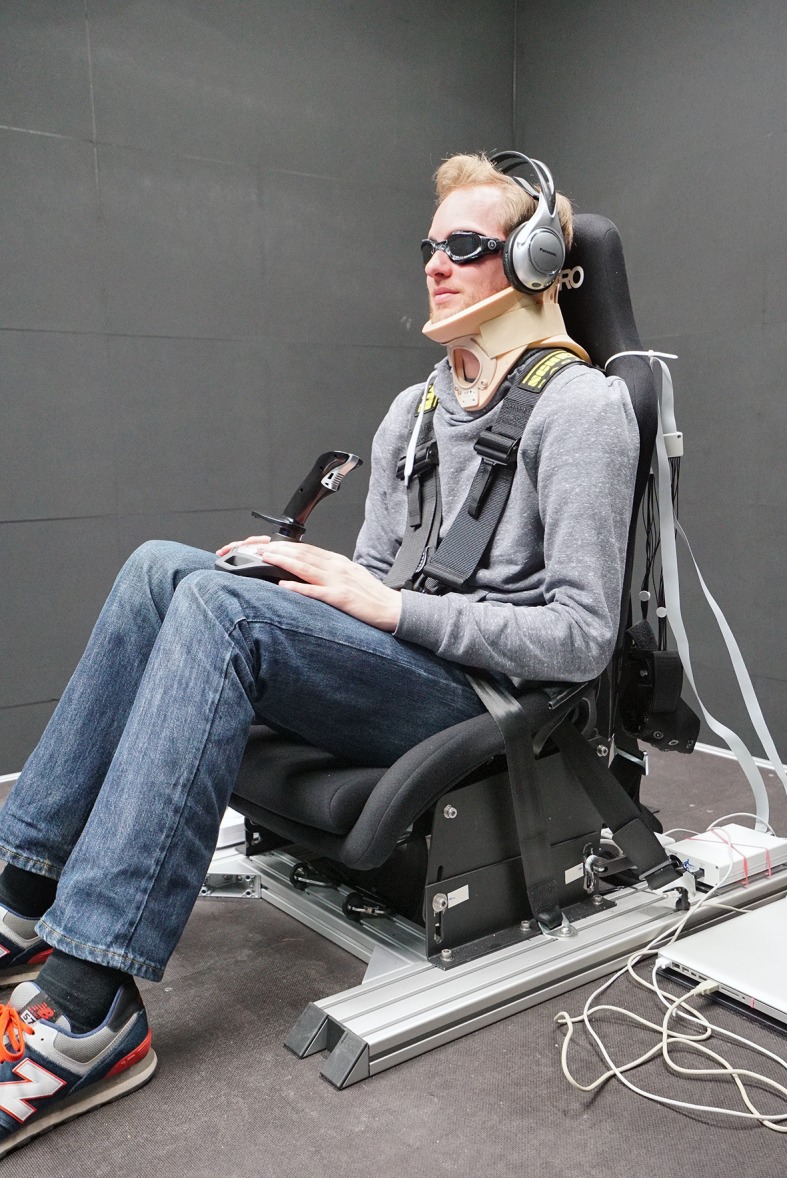
Experimental setup. The figure shows the platform seat mounted on top of the motion platform, with rails that allowed for small position adjustments prior to the experiment to ensure head-centred rotation (see text). Participants were blindfolded and secured with seat belts. White noise played through the headphones and ear plugs masked auditory cues from the simulator, goggles prevented visual motion cues and a neck brace helped maintaining the head stable.

We conducted the experiment using a 6 degrees-of-freedom hexapod motion system with six electric actuators (Bosch Rexroth eMotion 1500), which can reproduce yaw rotations of up to 41 deg/s within a range of 54 deg. Participants sat in a chair mounted on the platform and were secured with a 5-point safety harness (see Fig 1) and controlled the progress of the experiment with a button box with two active buttons. They wore light-proof goggles to preclude visual cues, and earplugs (SNR = 33) and headphones that, during stimuli presentation, played white noise to mask auditory cues from the actuators. They also wore a neck brace to help stabilize the head and maintaining an upright posture. Verbal communication between the experimenter and the participants was possible at any time during the experiment.

### Stimuli

**Fig 2:**
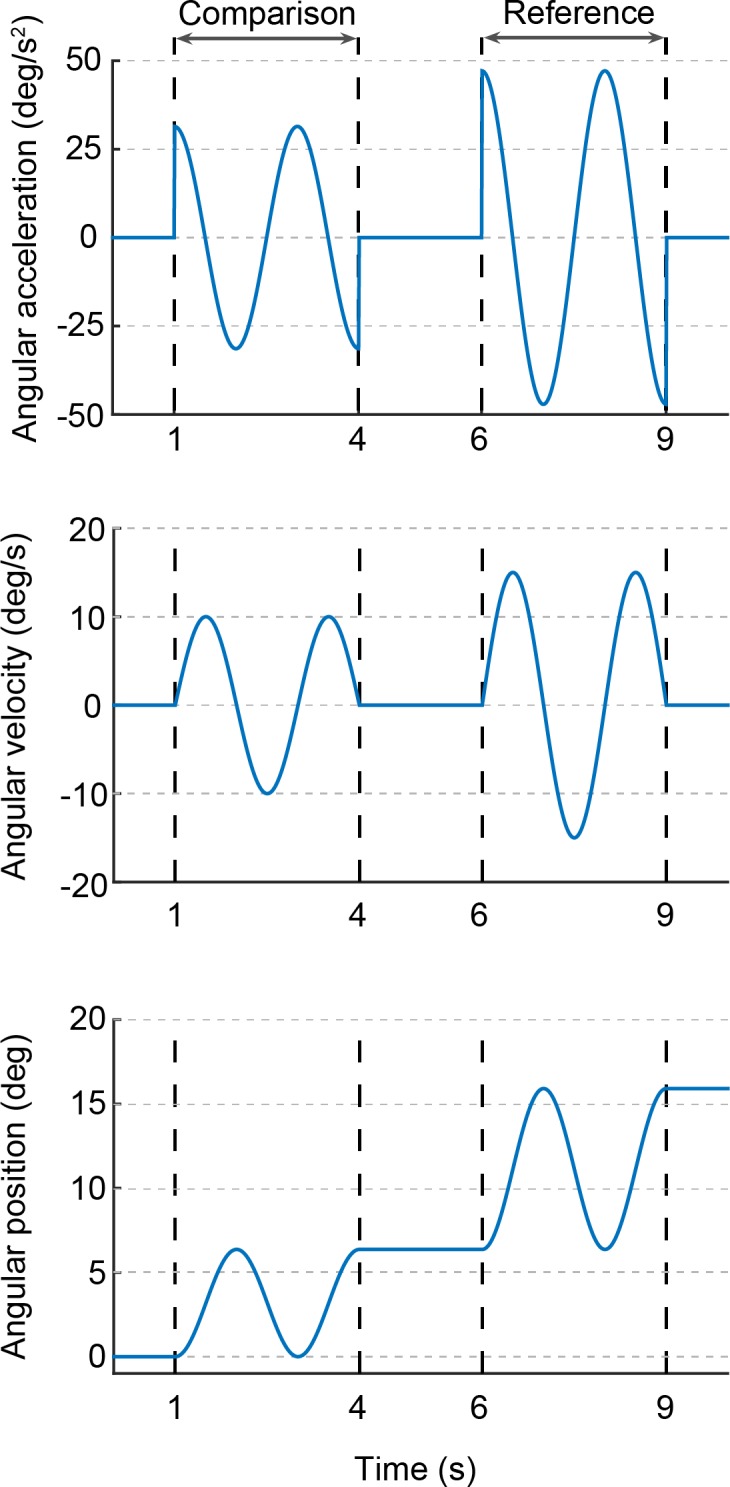
Stimulus profile. Acceleration, velocity and position traces of a typical trial composed of two stimuli lasting 3 s each. In this example, the reference stimulus, presented as second, has a velocity amplitude of 15 deg/s, while the comparison stimulus has a velocity amplitude of 10 deg/s.

Stimuli consisted of whole-body sinusoidal rotations around an earth-vertical yaw axis at 0.5 Hz (Fig 2). We adjusted the seat position to ensure that, during stimuli presentations, rotations were centred on the participant’s head. This was done by verifying the absence of centripetal accelerations on the vertical axis passing through the participant’s head using an inertial measurement unit (YEI 3-Space Sensor, 500 Hz) placed on top of a participant’s head.

During the entire duration of an experimental trial, we also commanded a constant level of randomly generated heave vibration to the platform. These vibrations were in the range of 4–8 Hz and had a root mean square (RMS) of approximately 0.1 m/s^2^ (comparable to the experience of driving on a bumpy road). They were unrelated to the yaw stimuli, and served two purposes. First, as suggested by Butler and colleagues [1], stimulus-unrelated vibrations could mask stimulus-related vibrations from the simulator, which are known to be amplitude dependent [31] and could introduce unwanted cues. Second, a background motion such as a vibration can increase perceptual thresholds [32,33]. Based on pilot results, inclusion of such vibrations in the present study could prevent a floor effect, which would occur if the discrimination task became too easy, obscuring the influence of stimulus duration on the DT.

### Procedure

Trials were initiated by participants through a button press and started 2 s later. Each trial consisted of two consecutive stimuli of equal duration and starting direction (left or right, randomly selected), separated by a 2 s pause (Fig 2). One of these stimuli, the ‘reference’ stimulus, always had an amplitude of 15 deg/s; the other stimulus, designated ‘comparison’, varied in amplitude across trials. One second after the second stimulus, the white noise and the vibrations stopped, and participants reported which of the two rotations felt stronger (i.e., higher velocity, acceleration and total displacement). Participants were explicitly asked to pay attention to the entire trial and only choose a response at the end of the second stimulus. The platform was then repositioned to the centre of its workspace with a constant velocity motion at 5.7 deg/s, which lasted on average 2.8 s. After repositioning, a beep sound played through the headphones indicated that the next trial could be initiated.

We presented trials according to the method of constant stimuli [13]. Comparison stimulus amplitudes ranged between 10 and 20 deg/s in steps of 1 deg/s, excluding amplitudes of 15 deg/s, and every comparison amplitude was repeated eight times. Stimulus duration could be 1, 2, 3 or 5 s. In total, each participant completed 320 trials in randomized order. Moreover, to avoid complications due to perceptual biases and motion aftereffect, we randomized between trials motion directions and reference/comparison presentation order. Data were collected over three sessions of approximately 45 minutes, with 5 minutes breaks every 15 minutes to avoid fatigue. Participants were only allowed to complete one session per day, and the entire data collection process took approximately 2 weeks. No session needed to be terminated because of fatigue or other reasons, and no participant reported symptoms of motion sickness.

### Data analysis

**Fig 3:**
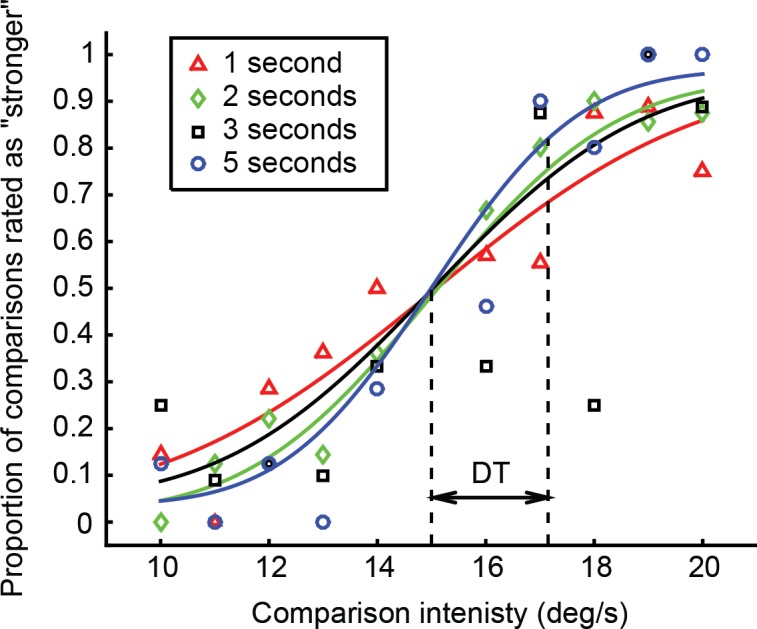
Psychometric functions. Typical psychometric functions (continuous lines) for one participant. Different marker shapes indicate different stimulus durations. Each marker corresponds to the proportion of responses (y axis) where the corresponding comparison intensity (x axis) was rated as stronger. The figure further illustrates the participant’s DT for the 5 s condition.

We separated the responses of every participant according to stimulus duration, and fitted four psychometric functions to analytically relate the stimulus amplitude to the probability of reporting the comparison stimulus as stronger. We modelled psychometric functions as Cumulative Normal distributions with two lapse parameters to account for stimulus unrelated errors, an improvement that can significantly increase the quality of the fit [34]. We performed the fitting by maximizing the likelihood function and constrained the lapse parameters to range between 0 and 0.05, and the mean of the Cumulative Normal distributions to equal the reference amplitude (15 deg/s). The standard deviation of the Cumulative Normal distribution that best fitted the data was arbitrarily chosen as the participant’s DT, since it reflects the slope of the psychometric function and therefore the discrimination capability of the participants. Typical psychometric functions for one participant are shown in Fig 3.

### Models of evidence accumulation

**Fig 4:**
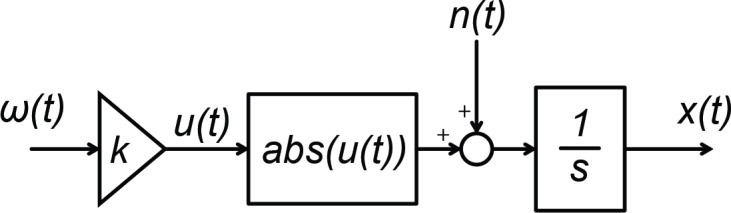
Block diagram representation of a DDM for accumulating sensory evidence. The physical stimulus omega(t) is first encoded by sensory organs (here represented by the gain K). Evidence of the stimulus property of interest (in this case stimulus intensity, computed by the abs() operation) is integrated over time (rightmost block, representing an integrator using Laplace notation). The noise signal n(t) represents physiological noise that is internal to perceptual processes and is responsible for trial-to-trial variability. In the leaky version of the DDM, a leaky integrator replaces the normal integrator (rightmost block).

We fitted two versions of the DDM to the experimentally measured DTs. In the first one (Fig 4), we modelled evidence accumulation as an integrator [20]. The model equation is:

$$\frac{dx(t)}{dt}=|K*\omega (t)|+n(t)$$ (1)

Where *K* relates the velocity of the dynamic yaw stimulus *omega(t)* to changes in the particle position *x(t)*, and *n(t)* is unit variance Gaussian noise representing physiological noise of the perceptual process.

Note that the model extracts the absolute value of the scaled input signal because participants had to focus on the stimulus intensity; therefore the velocity sign specifying whether rotations were to the left or to the right is irrelevant for the task.

In the second version of the DDM, we modelled evidence accumulation as a leaky integrator process:

$$\frac{dx(t)}{dt}=-R*x(t)+|K*\omega (t)|+n(t)$$ (2)

where *R* represents the rate at which information is ‘leaked’.

The initial condition for both models was *x(0) = 0*. We did not include a parameter for the noise variance, since assuming a non-unitary variance is equivalent to a simple rescaling of the other parameters [22,35].

Simulation of an experimental trial consisted in running the model two times, with *omega(t)* being once the reference and once the comparison stimulus. The model returns the stimulus with the larger *x(t)* as the stronger stimulus. In other words, the first stimulus is used to generate the decision bound for the second stimulus. We obtained the model parameters through numerical simulations by minimizing the sum of squared error between the experimentally measured DTs and DTs predicted through model simulation. The DTs predicted by the model were obtained using a Monte Carlo approach: the probability of reporting a comparison stimulus as stronger was computed by simulating the model 10000 times, an arbitrarily chosen number which represents a trade-off between computational cost and precision of the estimate. By solving an optimization problem we identify, for each stimulus duration, the comparison amplitude that is reported as stronger in 84.1% of the trials, consistent with the choice of using the standard deviation of the Cumulative Normal distribution as the participant’s DT. Finally, the DT prediction was obtained as the difference between this comparison amplitude and the reference amplitude. Note that, for the purpose of this simulation, we assume lapse parameters of 0.

### Stimulus noise model

As described above, finding the model parameters that best fit the experimental data requires an optimization routine that simulates the model using different comparison stimulus amplitudes. This routine needs to be able to freely select *any* stimulus amplitude, not just the ones employed in the study. Moreover, it requires complete knowledge of the dynamic physical stimulus to be simulated (indicated in Fig 4 as *omega(t)*). Ideally, inertial measurements of the platform motion should be employed, as they not only contain information on the motion commands, but also on any distortions introduced by the simulator (i.e., simulator-introduced noise). However, it is unfeasible to obtain recordings for every amplitude the optimization routine might select. We therefore opted for developing a stimulus noise model which allows for estimating the simulator response from the amplitude of the stimulus command.

The stimulus noise model is based on inertial recordings of the stimuli used in the actual experiment. The 5 s reference motion and every associated comparison motion were recorded ten times with an inertial measurement unit (YEI 3-Space Sensor, 500 Hz) aligned with the vertical axis passing through the participant’s head. Stimulus noise was then isolated using the procedure described in detail in [31]: recordings were low-pass filtered at 80 Hz, motions of equal amplitude and duration were averaged to isolate the deterministic component of the noise, and the corresponding motion command was finally subtracted. The RMS of every average trace, an indicator of the amount of noise, was found to depend on the intensity of the corresponding input command (F = 215, p<0.001). Therefore, noise traces were normalized so that every noise trace had an RMS of 1 m/s^2^, and averaged across stimulus intensity. This resulted in a noise “template” with duration of 5 s. A linear model was then fit so to predict the RMS of a general stimulus based on the amplitude of its sinusoidal motion command. The physical stimulus profile *omega(t)*, necessary to simulate the DDMs, was then obtained by adding to the sinusoidal motion command the noise template multiplied by its predicted *RMS* value. Noise templates for stimuli of shorter durations were obtained by truncating the original 5 s long noise template.

## Results

**Fig 5:**
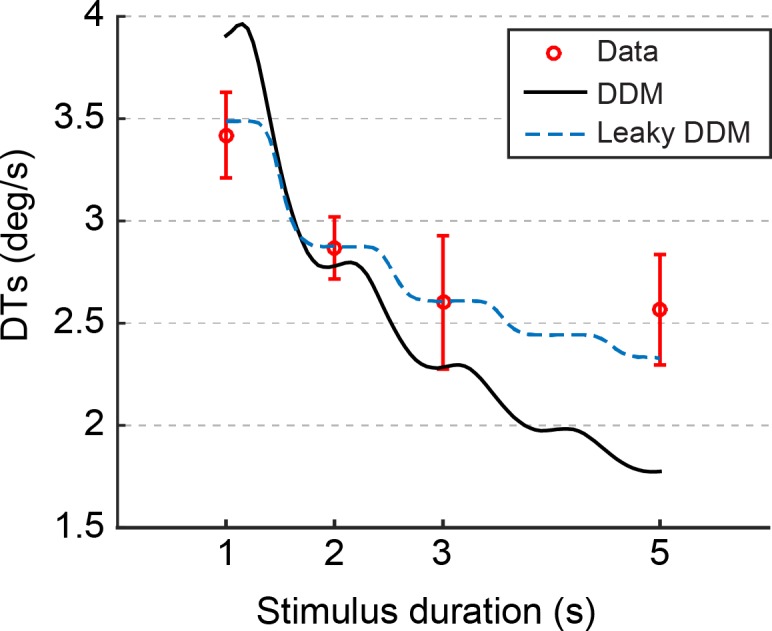
Comparison between model predictions and experimental data. Experimentally measured DTs (red circles) are well described by the DDM with leaky integration (blue line). The DDM with normal integration (black dashed line) provides a poor fit to the experimental data. Error bars represent ±1 standard error of the mean. For graphical purposes, models predictions were averaged over ten simulations of the stochastic models and smoothed over three consecutive values (moving average).

Averaged DTs are presented in Fig 5. As confirmed by linear regression analysis, DTs significantly decrease with the duration of the yaw stimuli (t(38) = 2.87, p = 0.007, r^2^ = 0.72). Over the tested range of stimulus durations, the highest DTs were measured for the 1 s condition (3.42 deg/s), while the lowest DTs were measured for the 5 s condition (2.57 deg/s).

No preference for a specific answer (1^st^ or 2^nd^) was observed between participants (t(39) = 0.54, p = 0.43), arguing against the emergence of motion aftereffect or velocity storage effect in the collected data.

As illustrated in Fig 5, the DDM with the normal integrator resulted in a poor fit to the experimentally measured DTs (r^2^ = -0.50, with *K* = 0.03 (deg/s)^-1^). Note that the negative r^2^ indicates how simply fitting the data with their average value would result in a better fit. A considerable improvement in goodness of fit (r^2^ = 0.96) was however obtained with the DDM that included a leakage term. The best fit for this model was obtained with *R* = 4.9 and *K* = 0.04 (deg/s)^-1^.

## Discussion

In this study, we measured DTs for discriminating two consecutive head-centred sinusoidal rotations of different amplitude. We found that stimulus duration has a significant effect on DTs, with lower DTs (i.e., better discrimination performances) for longer as compared to shorter stimulus durations. We further showed that a DDM with a leaky integration of sensory evidence can account for this effect. The following sections discuss the implications that methodological choices may have on the experimental results, the relation of the results to the literature and the tenability of DDMs.

### Methodological considerations

Before discussing the main findings, a number of clarifications on the experimental paradigm are necessary.

First, throughout the paper motion stimuli are described with respect to their velocity characteristic. This is a common choice in self-motion perception studies, since sinusoidal stimuli at 0.1–10 Hz evoke a perceptual response that is primarily velocity dependent [36,37]. Importantly, the stimuli employed in this study also contained a step-change in acceleration at their onset and offset, the amplitude of which depends on the stimulus peak velocity and could provide an unintended cue. However, although the presence of this additional cue could result in an overall reduction of DTs, it cannot account for the observed effect of stimulus duration since every employed stimulus, regardless of its duration, contains exactly two acceleration steps.

Second, when measuring DTs for yaw rotation, possible confounds may arise due to the velocity storage effect (i.e., a perception of rotation that persists after the physical rotational stimulus stops [38]) and due to motion aftereffects (i.e., the influence of previous stimuli on the perception of a subsequent stimulus [39]). Given the sinusoidal nature of the stimuli, the 2 s break between trials and the randomized presentation order, influences of these perceptual phenomena on the results are unlikely [16,39]. This is further supported by the observation that, over the entire study, no significant preference was observed between participants for a specific answer.

Finally, in studies on accumulation of sensory evidence in related fields, 2AFC experimental paradigms, where two stimuli are presented in concomitance, are more common than 2IFC paradigms, where two stimuli are presented one after the other (see e.g., [19,21,22,25]). In contrast to studies using a 2AFC paradigm, studies using a 2IFC paradigm require the assumption that participants can preserve the first stimulus (or at least its amplitude percept) in memory until a response is given. In the present study, the use of a 2IFC paradigm was dictated by the impossibility of measuring self-motion discrimination performances by presenting motion stimuli concurrently. Nevertheless, based on evidence on human information storage capabilities from the field of auditory perception [40], we find it reasonable to speculate that the intensity of the motion stimuli employed in the present study can be held in memory. Indeed, features of auditory stimuli can be “synthetized” and stored in what is commonly termed “synthetized auditory memory”, which can retain information for several seconds (up to 30s). In comparison, experimental trials of the present experiment lasted between 5 and 13 seconds. Due to these considerations, we did not include a memory mechanism in the DDMs, and we do not use these models for any inference on stimulus order effects or on the effect of a longer inter-stimulus break within trials. Future research should address the effects of varying the inter-stimulus break on DTs, thereby quantifying the capacity of the CNS to retain self-motion information. For a neural model integrating evidence accumulation, decision making and working memory in a 2IFC intensity discrimination task, we refer the interested reader to Machens and colleagues [41].

### Differential thresholds

A comparison of self-motion DTs for different stimulus durations was, to the best of our knowledge, never performed before. However, previous studies did measure DTs for supra-threshold yaw discrimination using a 2IFC experimental paradigm and 5 s long stimuli [6,9,16]. Nesti and colleagues [6] reported an average DT of 3.6 deg/s for a 15 deg/s reference stimulus amplitude, while Mallery and colleagues [16] measured an average DT of 2.26 deg/s for reference stimulus amplitudes of 20 deg/s. Results from the present study, with an average DT of 2.6 deg/s for a 5 s long stimulus with amplitude of 15 deg/s, are thus consistent with previous literature–minor discrepancies are attributable to inter-individual differences and to differences in the employed motion simulators. The higher DTs reported in the literature for higher yaw rotation intensities [6,10,16] should not surprise, due to the known relationship between DTs and stimulus intensity [6,16].

Previous works on absolute thresholds similarly report a dependency between the smallest perceivable constant angular acceleration and stimulus duration (Mulder’s law [36]). This relationship is explained by the mechanical deflection of the cupula within the semicircular canals of the vestibular system which, for smaller acceleration, requires more time to evoke a perceivable sensation [37]. In the present study, by employing suprathreshold stimuli we ensure that sensory information is available to the decision process throughout the whole stimulus.

The present work lays the ground for better informed comparisons between studies where different stimulus durations were used. For instance, observed asymmetries in the absolute thresholds of horizontal and vertical linear motions [32,42] have raised the question of whether DTs are similarly affected by motion direction. However, an indirect comparison of previously measured horizontal [17] and vertical [14] DTs for linear translations should also account for the differences in stimulus durations that exist between these two studies, so to prevent erroneous conclusions.

### Drift diffusion models

Since the seminal work of Ratcliff [19], DDMs have been mostly used to model accumulation of sensory evidence during decision tasks (see [23] for a review). Their ability to integrate information over time leads to an overall improvement in performances for longer stimulus presentations. This remains true when the integration process within the model includes a leakage term, but the rate at which evidence is accumulated is limited. For this reason, a DDM with leaky integration, in comparison to a DDM with normal integration, will always predict more moderate improvements for longer stimulus duration. In this study, we found that a DDM with a leaky integration mechanism accounts for the measured yaw DTs better than a DDM with normal integration (Fig 5). Despite leaky integration has been previously suggested and successfully included in DDMs for neurophysiological data [21], other studies did not require a leakage term to obtain high goodness of fit (see e.g., [22]). However, three important differences between previous behavioural studies and ours should be considered. First, the use of a 2IFC, rather than a 2AFC, experimental paradigm represents a qualitative change from the classical framework where DDMs are usually employed. The possibility that, despite the explicit instructions, participants committed to their decision before the end of the second stimulus could explain why performances are suboptimal, and even reconcile behavioural data with the DDM with normal integration. Second, while many DDMs implementations expect input stimuli with static properties [23], the present work employs a dynamic input, namely the sinusoidal time course of the rotational velocity signal, resulting in a non-linear accumulation of sensory evidence. This implies, for instance, that the velocity peaks are the most informative part of the stimuli, and that accumulating evidence when little sensory information is available might not improve performances (cf. Fig 5). These predictions could be tested in future studies by maintaining the first stimulus of fixed duration while the second is terminated by the participants. Finally, although DDMs have been extensively used in the study of perceptual mechanisms for many sensory modalities (e.g., visual or auditory), very little is known on whether they extend well to the field of self-motion perception. To the best of our knowledge, the only other work where DDMs were employed to model self-motion perception was conducted by Drugowitsch and colleagues [22], who successfully used a DDM to describe reaction times and accuracy in a multisensory heading discrimination task. Overall, the present work applies for the first time a DDM model to describe DTs for yaw rotations using a 2IFC paradigm. Even though the model provides evidence of the value of leaky DDMs in modelling self-motion perception, this evidence is not yet conclusive. More research is needed to fully establish DDMs as a tool for modelling the decision process underlying the perception of dynamic self-motion stimuli.

An interesting alternative to model the accumulation of sensory evidence is based on the idea that the number of velocity peaks, rather than the stimulus duration, determines the participants’ discrimination performances. Such strategy seems plausible when stimuli have dynamic properties: to achieve optimal performances, participants could decide to focus only on the velocity peaks, rather than the entire stimulus, as at the difference between reference and comparison is largest at the peaks. Although every participant reported to comply with the explicit instruction of paying attention to the whole stimulus, we cannot exclude that the decision process relied more on the peaks. The present experiment was not designed to address whether evidence is accumulated continuously or discretely; nevertheless, we attempted to gain more insight by testing our data against a Bayesian filter [43]. The implemented Bayesian filter updates a uniform prior expectation on the maximum stimulus velocity with normally distributed sensory readings. The sensory readings distribution is centred at a value that can be considered as a scalar representation of the true stimulus peak velocity. The standard deviation of the sensory readings is the only free parameter of the model. By updating the maximum velocity estimate at every stimulus peak (positive or negative), the model predicts increased accuracy for longer stimuli presentations, which is consistent with the observed data. However, as for the case of the DDM with normal integration, the model outperforms our participants, yielding a poor fit (r^2^ = -2.09). Future experiments should address whether and under what circumstances evidence accumulation for self-motion (i.e., dynamic) stimuli is sampled continuously or discretely, although it might not be trivial to experimentally discern between discrete and continuous, nonlinear accumulation of sensory evidence (cf. previous paragraph).

Experimental data on self-motion perception have been used in the past to develop mathematical models that describe the internal processes underlying self-motion perception [44–46]. The potential of these models to compute an internal representation (or percept) of the physical self-motion of an observer is of great value for a variety of applied fields, such as for example vehicle motion simulation [47–49] or the diagnosis of clinical disorders [50]. Nevertheless, these perception models are of a deterministic nature, and cannot therefore capture the variability of individual responses. The block diagram from Fig 4 suggests a possible improvement by including a random noise term after the transduction of the physical stimulus (modelled in our case a simple gain, more commonly by a transfer function describing the dynamics of the sensory organs). A similar idea was suggested by Bigler and Cole [51] to model motion detection thresholds. These solutions introduce a probabilistic component which will allow perception models to make predictions on both the average expected percept as well as its variability, thus accounting for experimentally observed perceptual phenomena such as inter-trial variability.

## Supporting Information

S1 Data Experimental data. (ZIP)
